# Masking Policies at National Cancer Institute–Designated Cancer Centers During Winter 2023 to 2024 COVID-19 Surge

**DOI:** 10.1001/jamanetworkopen.2024.24999

**Published:** 2024-07-31

**Authors:** Michael Hoerger, Dulcé Rivera, Brenna Mossman, Birney Sherard, Tristen Peyser, Taylor M. Alcorn

**Affiliations:** 1 Tulane Cancer Center, Tulane University, New Orleans, Louisiana; 2 Department of Psychology, Tulane University, New Orleans, Louisiana; 3 Department of Medicine (Hematology and Oncology), Tulane University, New Orleans, Louisiana; 4 Department of Palliative Medicine and Supportive Care, University Medical Center, New Orleans, Louisiana

## Abstract

This cross-sectional study examines variation in masking policies at National Cancer Institute (NCI)–designated cancer centers during the winter 2023-2024 COVID-19 surge.

## Introduction

Although people with cancer have above-average risk of COVID-19 vaccine antibody nonresponse, breakthrough infections, hospitalizations, long COVID, infection-associated treatment delays, and mortality,^1,2^ health care masking policies remain contentious. Surveillance data suggest the winter 2023 to 2024 surge had the second highest peak of COVID-19 transmission in the US.^3,4^ We assessed masking policies at National Cancer Institute (NCI)–designated cancer centers near the midpoint of the winter 2023 to 2024 US COVID-19 surge and examined variation by region and proxy indicators of quality^5^: NCI designation duration, funding, and care rankings.

## Methods

In this cross-sectional study, we reviewed the websites of all NCI-designated clinical cancer centers to analyze COVID-19 policies on January 15, 2024. This date was selected based on forecasting models suggesting the date to be near the surge’s midpoint, estimated in hindsight to be January 9, 2024.^4^ Two raters (including D.R., B.M., B.S., T.P., T.M.A.) identified whether any universal masking requirements (eg, all staff and visitors must mask in designated areas) existed. Raters also recorded the locations with requirements, whether any nonuniversal masking requirements (eg, mask if symptomatic) existed, and whether the websites indicated or linked directly to current COVID-19 policies. At least 1 rater called each center within 72 hours to verify policies and, in rare situations of uncertainty or inconsistency, asked to speak to an individual who could provide definitive policy information. In accordance with the Common Rule, this study was exempt from ethics review and informed consent requirement because it was not considered human participant research. We followed the STROBE reporting guideline.

Frequencies and percentages summarized the descriptive statistics. Exploratory analyses used χ^2^ and Kendall tau to examine whether Census region (Northeast, Midwest, South, West), ongoing NCI designation duration (quintiles), cumulative NCI-designated program funding (P30 grants) (quintiles), and *US News & World Report* oncology care rankings (quintiles) were associated with having a universal masking policy in at least some designated areas (yes or no).

Two-sided *P* < .05 indicated statistical significance. Analyses were conducted in SPSS 28.0.1.1 (IBM).

## Results

COVID-19 policies were confirmed at all 67 patient-serving NCI-designated cancer centers. As shown in Table 1, 28 cancer centers (41.8%) required universal masking in at least some clinical areas, with 12 (17.9%) requiring universal masking in all areas. Only 14 (20.9%) had accurate up-to-date policies flagged on the home page of their websites. In 8 cancer centers (12.0%), policies posted on websites differed from those noted by telephone. Cancer centers were more likely to require universal masking in at least some areas if they were located in the Northeast (11 [78.6%]), had longer NCI designation duration (first quintile: 10 [83.3%]), had more program funding (first quintile: 11 [84.6%]), or had a higher care ranking (first quintile: 11 [84.6%]) (Table 2).

**Table 1.** Masking Policies at National Cancer Institute–Designated Cancer Centers During the US COVID-19 Surge in Winter 2023 to 2024^a^

Characteristic	Cancer Center, No. (%) (N = 67)
Universal masking precautions in any area of the cancer center	
No	39 (58.2)
No requirement	28 (41.8)
Only required for staff	4 (6.0)
Required or recommended if symptomatic	7 (10.4)
Yes	28 (41.8)
Required in certain locations, but not specified online or by telephone	6 (9.0)
Required in specified locations	10 (14.9)
Required in all areas, unless doffing was temporarily necessary	12 (17.9)
Current COVID-19 policies provided or linked on cancer center website	
No, not provided	48 (71.6)
No, provided but outdated	5 (7.5)
Yes, up-to-date	14 (20.9)
Inconsistencies between cancer center website and telephone information	
Stronger precautions indicated by telephone vs website	4 (6.0)
Weaker precautions indicated by telephone vs website	4 (6.0)

^a^ Telephone information was used when there were discrepancies in precaution policies from the website. Website data were collected on January 15, 2024, and telephone data were collected within 72 hours of this date. All data were collected shortly after the midpoint of the surge on January 9, 2024.

**Table 2.** Proportion of National Cancer Institute–Designated Cancer Centers With Universal Masking Precautions

Characteristic	No./total No. (%) (N = 67)	*P* value
US Census region		
Northeast	11/14 (78.6)	.01^a^
Midwest	3/14 (21.4)	
South	8/23 (34.8)	
West	6/16 (37.5)	
NCI designation duration, quintile		
First, longest duration	10/12 (83.3)	<.001
Second	8/13 (61.5)	
Third	5/15 (33.3)	
Fourth	3/14 (21.4)	
Fifth, shortest duration	2/13 (15.4)	
NCI-designated program funding (P30 grants), quintile^b^		
First, most funded	11/13 (84.6)	<.001
Second	6/14 (42.9)	
Third	4/13 (30.8)	
Fourth	6/14 (42.9)	
Fifth, least funded	1/13 (7.7)	
*US News & World Report* ranking, quintile^c^		
First, highest ranked	11/13 (84.6)	.02
Second	4/14 (28.6)	
Third	4/13 (30.8)	
Fourth and Fifth, lowest ranked	9/27 (33.3)	

Abbreviation: NCI, National Cancer Institute.

^a^ A post hoc contrast indicated that universal masking precautions were more common in the Northeast than other regions (78.6% v 32.1%; *P* = .002).

^b^ NIH RePORTER funding records covered 1985 through February 2024.

^c^ When a cancer center had more than 1 affiliated clinic in the rankings, the higher of the rankings was used. Bottom quintiles were combined because none of the institutions were ranked in the top 50 of the *US News & World Report*.

## Discussion

Highlighting clinical equipoise, 41.8% of NCI-designated cancer centers required universal masking in at least some clinical areas during the winter 2023 to 2024 COVID-19 surge. Such policies were more common in the Northeast, despite higher transmission in the South and Midwest.^3^ Longer NCI designation, more program funding, and higher care rankings were also associated with having universal masking policies. With 8 waves of elevated COVID-19 transmission,^3,4^ health care system–acquired COVID-19 infections are highly preventable, with debates surrounding prevention pros and cons.^6^ Cancer centers with masking policies should delineate the data and decision-making models underlying their policy to inform other centers considering their own policies.

Study limitations include a focus on a time point in the COVID-19 pandemic, in the US, and at academic cancer centers with NCI designation; thus, findings may have limited generalizability to other contexts. More research funding and studies are needed to examine the implications of mitigation policies for infection rates among patients and medical personnel, treatment discontinuities, hospitalizations, long COVID, and mortality. Although contentious, universal masking precautions were common at NCI-designated cancer centers during the winter 2023 to 2024 surge, especially at more established, better-funded, and higher-ranked centers.

## Notes

Supplement.
Data Sharing Statement

## References

[1] Hoerger M, Gerhart J, Swartz MC. Variability in COVID-19 vaccine response among people with cancer: what health care strategy best protects the vulnerable? JAMA Oncol. 2023;9(2):177-179. doi:10.1001/jamaoncol.2022.5874 36547943

[2] Llanos AAM, Ashrafi A, Ghosh N, . Evaluation of inequities in cancer treatment delay or discontinuation following SARS-CoV-2 infection. JAMA Netw Open. 2023;6(1):e2251165-e2251165. doi:10.1001/jamanetworkopen.2022.51165 36637818 PMC9856904

[3] Centers for Disease Control and Prevention, National Wastewater Surveillance System. Wastewater COVID-19 national and regional trends. Accessed June 1, 2024. https://www.cdc.gov/nwss/rv/COVID19-nationaltrend.html

[4] Hoerger M. Pandemic mitigation collaborative—COVID-19 forecasting model. Accessed June 1, 2024. https://www.pmc19.com/data

[5] Boffa DJ, Mallin K, Herrin J, . Survival after cancer treatment at top-ranked US cancer hospitals vs affiliates of top-ranked cancer hospitals. JAMA Netw Open. 2020;3(5):e203942-e203942. doi:10.1001/jamanetworkopen.2020.3942 32453382 PMC7251445

[6] Klompas M, Baker MA, Rhee C. Is nosocomial SARS-CoV-2 still worth preventing? JAMA Netw Open. 2023;6(11):e2344704-e2344704. doi:10.1001/jamanetworkopen.2023.44704 37948088

