# Supplementary material for: Masking Policies at National Cancer Institute–Designated Cancer Centers During Winter 2023 to 2024 COVID-19 Surge
Source: JAMA Netw Open. 2024 Jul 31;7(7):e2424999. doi: 10.1001/jamanetworkopen.2024.24999 (PMC11292445; doi:10.1001/jamanetworkopen.2024.24999)
Supplement: Supplement. — Data Sharing Statement [file jamanetwopen-e2424999-s001.pdf]

Data Sharing Statement

Hoerger. Masking Policies at National Cancer Institute–Designated Cancer Centers During Winter 2023 to 2024 COVID-19 Surge. *JAMA Netw Open*. Published August 01, 2024. doi:10.1001/jamanetworkopen.2024.24999

Data
Data available: No

Additional Information
Explanation for why data not available: The data set includes information related to policies, funding records, and other quality metrics for individual cancer centers that we regard as sensitive. We prefer to handle such data requests on an individual basis, rather than posting online.
